# Cystic Fibrosis in a Female Infant with Cardiac, Ocular, and Musculoskeletal Anomalies

**DOI:** 10.1155/2015/379018

**Published:** 2015-11-29

**Authors:** Azhar Farooqui, Susan Gamal Eldin, Muna Dawood Ali, Ali AlTalhi, Ahmad AlDigheari

**Affiliations:** ^1^College of Medicine, Alfaisal University, Riyadh 11533, Saudi Arabia; ^2^Department of Pediatrics, Security Forces Hospital, Riyadh 12625, Saudi Arabia

## Abstract

Cystic fibrosis (CF) remains the most common hereditary disease in the western population. Its concomitant presence with other congenital abnormalities is a rare phenomenon with very little documentation. In this case report we describe a case of cystic fibrosis in a female infant with cardiac, ocular, and musculoskeletal abnormalities. A brief literature review is also provided.

## 1. Introduction

Cystic fibrosis remains as the commonest hereditary disorder in the western population. The incidence of this autosomal recessive disorder has been estimated to be around 1 in 4100 live births in the United States [1]. Pulmonary symptoms are often the mode of presentation at the time of diagnosis with up to 95% of the affected patients observed to have recurrent pulmonary infections [2]. Gastrointestinal complications are a common finding; pancreatic insufficiency remains the commonest reported complication. Diagnosis of cystic fibrosis before the age of 14 years has been associated with a greater risk of pancreatic exocrine insufficiency [3].

Association of cystic fibrosis with other diseases or congenital anomalies is rare. In Saudi Arabia, only a few of these associations including sickle cell anemia, Ehler-Danlos syndrome (EDS), insulin dependent diabetes mellitus, and congenital adrenal hyperplasia have been documented from a single center experience [4]. In this case report, the authors describe a case of cystic fibrosis with multiple cardiac, ocular, and musculoskeletal abnormalities. A brief literature review is also presented.

## 2. Case

A 3-month-old baby girl presented to our emergency department (ER) with cough, shortness of breath, and difficulty in breathing as noticed by the mother. The baby is a product of spontaneous pregnancy, delivered at full-term via cesarean section at her local hospital due to breech presentation. The baby was admitted in the neonatal intensive care unit (NICU) of her local hospital immediately after delivery due to possibility of perinatal asphyxia; however, she was discharged in stable condition without complications. Further history revealed that the patient was admitted to her local hospital at the age of one month as a case of bronchiolitis, received supportive therapy, and was discharged in stable condition.

**Figure 1:**
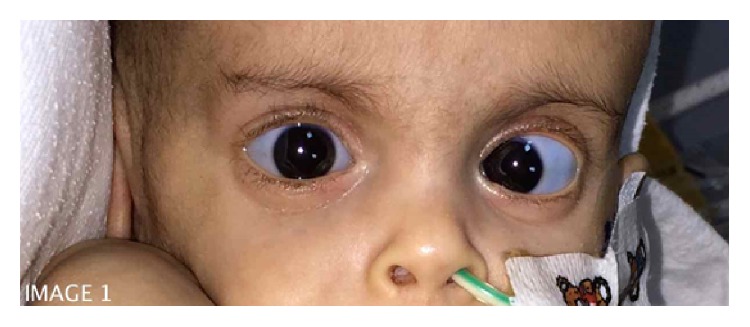
An image of the patient to demonstrate the blue sclera and left eye esotropia.

**Figure 2:**
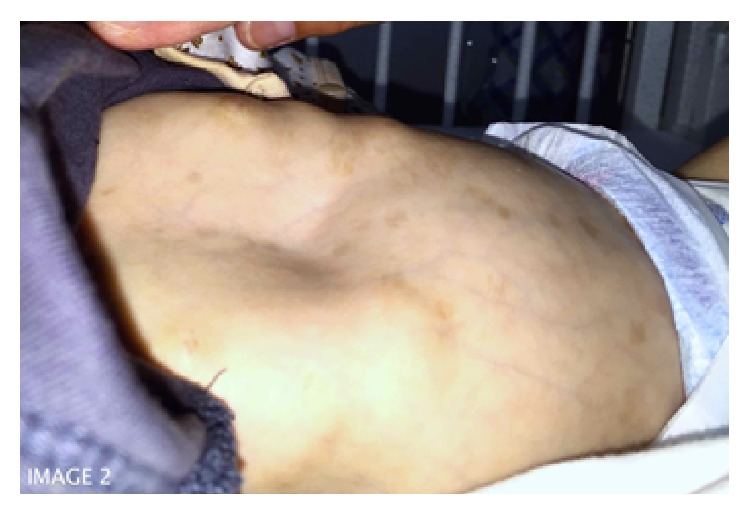
An image of the patient demonstrating pectus excavatum.

Upon presentation to our ER, patient was vitally stable with 98% oxygen saturation on room air; weight was 2.56 kg (<5th percentile), head circumference 32 cm (<5th percentile), and height 52 cm (<5th percentile). Cardiac examination demonstrated preserved 1st and 2nd heart sounds with a pan-systolic murmur at the left sternal border. Chest examination revealed decreased air entry bilaterally, with bilateral crepitation. Central nervous system examination demonstrated generalized hypotonia (upper limbs greater than lower limbs) with presence of head lag. The patient had blue sclera with left eye esotropia (Figure 1), thin ears, arched palate, and depressed nasal bridge. There was a significant pectus excavatum (Figure 2). Also appreciated was a transparent appearing skin with extremely prominent scalp veins. There was increased elasticity in the skin over the chest (2-3 cm) and increased mobility in the thumb with a positive thumbs sign. However, there was no other joint laxity or hypermobility in axial or other peripheral joints. Patient was admitted as a case of community acquired pneumonia and failure to thrive. Appropriate antibiotics and supportive managements were initiated.

Blood work-up demonstrated a positive nasopharyngeal culture for* Pseudomonas aeruginosa*. Sweat chloride test was carried out which was positive in two different sets with results 90 and 137 mmol/L, respectively (range 3–60 mmol/L). Stool test for pancreatic elastase demonstrated exocrine pancreas insufficiency. Chromosomal study was unremarkable. Blood samples were sent for mutations in cystic fibrosis genes to the United States, which were negative. However, the diagnosis of cystic fibrosis could not be ruled out due to lack of data on the sensitivity of the genetic study in the Saudi population, with the laboratory suggesting a clinical correlation. A diagnosis of cystic fibrosis was made after careful review of the patient's history and clinical presentation, positive sweat chloride tests, signs of pancreatic insufficiency, and multiple bouts of upper respiratory infections growing* Pseudomonas aeruginosa* on blood cultures.

Echocardiogram demonstrated bicuspid aortic valve with mild to moderate aortic stenosis, dilated ascending aorta, dilated superior vena cava, mild left ventricular hypertrophy, and a moderate restrictive patent ductus arteriosus with left to right shunt (PDA). Ultrasound of the brain, ultrasound of the abdomen, and CT scan of the brain were all unremarkable.

The patient was discharged home in a stable condition with pancreatic amylase supplementation, vitamin supplementation, fluticasone and Ventolin inhalers, and prophylactic antibiotics with close outpatient follow-up.

## 3. Discussion

Acute cardiac failure in pathologic myocardial fibrosis often characterizes cardiac involvement of cystic fibrosis in infants. Cor pulmonale as a result of chronic hypoxemia with resultant effect on the pulmonary vasculature is the dominant and a more serious presentation of cardiac involvement in older affected individuals [5, 6]. However, there is very little documentation regarding congenital cardiac anomalies in patients with cystic fibrosis and their effect in the prognosis of these patients [4]. From the Arab population, only Banjar and Mogarri [7] have reported a single case of twins affected by cystic fibrosis with concomitant cardiac anomalies; one sibling was affected by an atrial septal defect, which was treated surgically, while the other sibling was affected by a ventricular septal defect which closed spontaneously. In our case, there is a bicuspid aortic valve with mild to moderate stenosis of the aortic valve. Also associated is a PDA with left to right shunt, which may accelerate cardiac dysfunction in such cases. Ascending aortic dilatation may be explained by an underlying collagen defect as described below.

Although extremely rare, collagen fiber disorders such as EDS have also been reported to occur in patients with cystic fibrosis. To date, there are only two published studies describing cases of cystic fibrosis with classical concomitant presentation of EDS. One was an association of type 6 (kyphoscoliosis type) where two siblings from consanguineous Turkish parents were observed to have marfanoid habitus, generalized hypotonia, progressive kyphoscoliosis, and joint dislocations [8]. The other case had type 3 (hypermobility type) EDS with hypermobility of all joints and elastic skin [4]. In our case there is an element of possible collagen defect disorder though it does not fit the typical clinical criteria for the 10 different types described in the literature. The patient had blue sclera, which is a feature of the rare spondylocheirodysplasia EDS [9]. The patient also had pectus excavatum, generalized hypotonia, prominent scalp veins, and thin, stretchable skin. However, apart from hypermobility in the thumb joints, with a positive thumbs sign, there was no joint laxity in other axial or other distal joints.

The incidence of cystic fibrosis in the Saudi population has been estimated to be around 1 : 4200 live births [10]. It has been associated with various other anomalies in the Saudi population. Banjar [4] described an association of cystic fibrosis with sickle cell disease, insulin dependent diabetes mellitus, and congenital adrenal hyperplasia. No other publications can be found in the literature to demonstrate association of congenital anomalies with cystic fibrosis in the Arab population.
